# Saving More Teeth—A Case for Personalized Care

**DOI:** 10.3390/jpm5010030

**Published:** 2015-02-16

**Authors:** Alexandre R. Vieira, Katelyn M. Hilands, Thomas W. Braun

**Affiliations:** Department of Oral Biology, 614 Salk Hall, School of Dental Medicine, University of Pittsburgh, 3501 Terrace Street, Pittsburgh, PA 15261, USA; E-Mails: kmh191@pitt.edu (K.M.H.); twb3@pitt.edu (T.W.B.)

**Keywords:** dental caries, periodontal diseases, tooth loss, gingivitis, personalized medicine, interleukins, diabetes, smoking, cardiovascular diseases

## Abstract

Background: Certain risk factors such as tobacco use, diabetes, genetic variations on the IL1 gene, and other inflammatory conditions are hypothesized to predict tooth loss in patients treated in a large medical center. Tooth loss trends are hypothesized to be greater in patients with more risk factors. Methods: DNA samples for 881 individuals were taken from the Dental Registry and DNA Repository at University of Pittsburgh School of Dental Medicine. Clinical data for all 4137 subjects in the registry were also available. SNP genotyping was performed on the samples for IL1α (rs1800587) and IL1β (rs1143634). IL1 positive status was determined as having one or more of the recessive alleles for either SNP. Tooth loss status was determined based on dental records and data gathered for age, sex, ethnicity, and self-reported medical history. Various statistical analyses were performed on the data including genetic association analysis by the PLINK software, chi-square, Mann-Whitney U, and ANOVA tests to determine significance. Results: Tooth loss averages increased with age by all risk factors (smoking, diabetes, hypertension, and interleukin genotypes; *p* = 4.07E-13) and by number of risk factors (*p* = 0.006). Increased tooth loss is associated with age and number of risk factors including diabetes, tobacco use, IL1+, and cardiovascular disease. Conclusion: These trends suggest that older patients and those with more risk factors should seek further preventive care to reduce future tooth loss.

## 1. Introduction

In dentistry, the traditional claims of “everyone should go to the dentist twice a year” and/or “everyone should brush their teeth three times a day” are actually not based on evidence. They are supported by common sense and the perception that these guidelines appear to be right for most of people. The selection of an appropriate recall interval for patients is quite difficult [1] since there are so many factors that impact the two most common bacteria-mediated diseases that affect teeth.

Recent years have brought a renewed interest in the impact of oral health on overall health. Gum inflammation (periodontal disease) has been linked to a number of adult diseases, which include cardiovascular conditions and premature births. The two common bacteria-mediated diseases of the mouth, dental caries and periodontal diseases, continue to be highly prevalent and are the leading causes for tooth loss. Tooth loss is associated with poorer quality of life. Evidence shows that 80% of the burden of these diseases is confined to 25% of the population. So it would be most beneficial to focus public health efforts in identifying and implementing more aggressive preventive strategies for the segment of the population which needs the most care [2]. When more than 5000 subjects had their visits to the dentist contrasted with their levels of tooth loss, individuals who smoked, were diabetic, and carried specific interleukin 1 gene alleles benefited more from visiting the dentist twice a year. On the other hand, no additional benefit was seen for individuals without any of the above-mentioned risk factors [3].

Pittsburgh is the largest city adjacent to one of the poorest areas in the USA, Appalachia. The Appalachian mountain range extends across 13 states in the United States from New York to Mississippi. Positive core values associated with the region include strong sense of community, strong family support systems and social ties, religious affiliation, pride in self and family, independent self-reliance, the importance of justice, loyalty, religion, faith in god, strong work ethic, trustworthiness, and a feeling of belonging in the mountains. More problematic stereotypes and/or cultural norms are also associated with the region, such as fatalistic outlook, distrust of outsiders, and distrust of formalized medical systems. Independent from these data, quantitative data clearly show socio-economic indicators are much worse for the communities in the Appalachian region compared to those in the rest of the United States (reviewed in [4]). In regard to health indicators, Pittsburgh reflects what is found in the Appalachian region, and the population treated at the University of Pittsburgh Medical Center has some of the worst health indicators in the country, which makes Pittsburgh a perfect laboratory for studying disease risks. We keep a registry of patients in our School of Dental Medicine, where clinical information is linked to a biological sample of consented individuals. This resource (The Dental Registry and DNA Repository project) is available for supporting hypothesis driven work that requires careful dental clinical information. Details of this project were described previously [5]. Here we report the analyses of the population participating in the registry and measured tooth loss as the outcome of interest aiming the identification of risk factors that may guide a preventive strategy that will reduce tooth loss and increase tooth longevity.

## 2. Results

An analysis of the self-reported medical history adjusted by sex, age, and ethnic background indicated that individuals who smoked, had diabetes, or cardiovascular diseases had statistically more tooth loss (Table 1). Therefore, besides diabetes status, smoking habits, and interleukin 1 genotypes, we added hypertension as a fourth risk factor for analyses. We initially selected 881 adult subjects out of the 4137 with complete periodontal examinations and evaluated tooth loss based on smoking, diabetes, having interleukin 1 risk alleles, and hypertension. The data showed that increasing tooth loss levels correlated with accumulating risk factors (Table 2). 14.5% of the subjects studied had no risk factors and more teeth in their mouths. Since all those variables are associated with aging, we analyzed the data again by dividing the sample in adults younger than 50 years of age and older than 50 years of age. The same trend can be seen in both groups however the trend of increased tooth loss as one accumulates risk factors is much clearer in people younger than 50 years of age (Table 2). We repeated these analyses with all 4137 subjects without considering the interleukin 1 genotypes (Table 2). Despite seeing the same trend persist, the number of individuals defined as having no risk increased from 14.5% to 54% indicating that the genomic analysis is necessary for correctly defining individual risks in this case.

**Table 1:** Tooth loss average by risk factors (regression analyses adjusted by age, sex, and ethnic background) of 4,137 individuals from the University of Pittsburgh School of Dental Medicine Dental Registry and DNA Repository.

Risk Factor	Exposure		*p*-value
	Yes	No	
Smoking	10.82	8.76	0.02
Diabetes	11.85	9.03	<0.01
Cardiovascular Diseases (high blood pressure + stroke)	12.19	8.99	<0.01

Note: Mean age of patients in the registry is 48 years (standard deviation 17 years, patient’s ages ranging from 6 to 94 years), 53% of patients are males, 47% females; 73% are White, 21% Black, and the remaining 6% are comprised by other groups.

## 3. Discussion

Pittsburgh is the largest city in the Appalachian region of the United States, and one of the poorest in the country. Pittsburgh has had fluoridated water since 1953; however, nearly half of the children in Pittsburgh between six and eight have had cavities according to a 2002 State Department of Health report [6]. More than 70% of 15-year-olds in the city have had cavities, the highest percentage in the state. Close to 30% of the city’s children have untreated cavities. That is more than double the Pennsylvania state average of 14%.

The most common reasons for tooth loss are dental caries and periodontal diseases. In our sample, particularly for the group under 50 years of age, dental caries is the main reason individuals lost their teeth. Interleukin 1 composite genotypes have been investigated for a role in periodontitis, both looking at the progression of disease, as well as treatment outcomes, but results are inconclusive and the suggestion of performing systematic genetic testing for periodontitis has been challenged (reviewed by [7]). Our data suggest that interleukin 1 genotypes may be useful and the main difference is that we have measured tooth loss in contrast to studies in the periodontal literature that measured other clinical signs of periodontal diseases such as clinical attachment loss. Like having diabetes, smoking, or having high blood pressure, genotype results should be interpreted as another risk factor that may modify risk for tooth loss in the future. Similar to high blood pressure that cannot be attributed to directly contribute to either tooth loss due to dental caries or periodontal diseases, interleukin genotypes studied here are not necessarily causing dental caries or periodontal diseases but serving as markers for risk to tooth loss. Possibly, genetic variation of other inflammatory genes that have been studied for dental caries or periodontal diseases can provide similar information regarding risks for tooth loss.

**Table 2:** Accumulated Risk Factors and Tooth Loss.

Number of Teeth Lost on Average	Without Considering Age (N = 881)	Considering Age (N = 881)		Without Considering Genomics Data (N = 4137)
		Younger than 50 years	50 Years or Older	
No Risk Factors	5.79 (N = 128)	3.45 (N = 75)	9.09 (N = 53)	3.6 (N = 2,229)
One Risk Factor	9.28 (N = 323)	7.17 (N = 174)	11.74 (N = 149)	9.29 (N = 1,418)
Two Risk Factors	10.87 (N = 242)	10.49 (N = 105)	11.18 (N = 137)	12.29 (N = 430)
Three Risk Factors	11.45 (N = 78)	10.11 (N = 19)	11.88 (N = 59)	15.98 (N = 60)
Four Risk Factors	13.1 (N = 10)	–	13.1 (N = 10)	–
Any Number of Risk Factors	10.19 (N = 753)	8.52 (N = 298)	11.58 (N = 355)	10.18 (N = 1,908)
*p*-value (calculated comparing individuals with no risk factors and any number of risk factors)	<0.01	<0.001	0.05	<0.000001

Risk factors are smoking habits, diabetes status, blood pressure level, and being a carrier of at least one variant allele for either interleukin 1 genotypes for markers rs1800587 and rs1143634.

In aggregate, these data indicate a model of personalized dentistry in which individuals receive a customized schedule for their periodic dental visits is warranted. A portion of the population that “tests” positive for a higher risk for tooth loss based on smoking and diabetes status, blood pressure levels, and interleukin 1 genotypes should visit the dentist more often, as much as four to six times per year. Individuals can potentially be tested as early as adolescence since the pathological processes and risk factors associated with the development of cardiovascular diseases have been shown to begin in childhood, and similarly, the incidence of type 2 diabetes reported in children has increased substantially (reviewed in [8]). In contrast, individuals who tested as having lower risk for tooth loss may not need to visit the dentist twice a year, and perhaps can be seen as infrequently as every other year or once every three years. Previous data show that individuals at low risk for tooth loss do not have any additional benefits from visiting the dentist twice a year as currently recommended [3]. The revision of current private insurance coverage schedules and government-assisted reimbursements would likely save an unprecedented amount of resources by implementing these guidelines. This can be achieved by avoiding the costs of unnecessary dental visits and also by preventing more expensive dental visits later in life for treatments of extensively destroyed teeth or lost tooth supportive structures that have poorer prognosis and short longevity.

## 4. Experimental Section

At the time of these analyses, 4137 subjects from the University of Pittsburgh School of Dental Medicine Dental Registry and DNA Repository (University of Pittsburgh Institutional Review Board (IRB) approval #0606091) were available for study. Starting in September of 2006, all individuals that seek treatment at the University of Pittsburgh School of Dental Medicine have been invited to be part of the registry. These individuals give written informed consent authorizing the extraction of information from their dental records. Tooth loss was defined as the total number of teeth extracted due to caries or periodontal disease. Teeth extracted due to orthodontic reasons, trauma, or congenitally missing did not contribute to the total count per subject. Longitudinal data to estimate tooth loss overtime was not available for all individuals in the population and tooth loss was defined according to the most recent visit to our clinics. The average age of the population is 48 years and we decided to analyze further the data based on individuals younger and older than 50 years of age with the assumption that individuals younger than 50 years are less impacted by periodontal diseases and most of their tooth losses can be attributed to dental caries. Further stratification did not provide additional insight [9]. An analysis of the self-reported medical history adjusted by sex, age, and ethnic background indicated that individuals who smoked, had diabetes, or cardiovascular diseases had statistically more tooth loss (Table 1). We did not include socioeconomic status indicators because it is well established lower socioeconomic status is associated with higher incidence of both dental caries and periodontal diseases. Also, the majority of individuals that participate in the registry belong to groups of lower socioeconomic status. Therefore, besides diabetes status, smoking habits, and interleukin 1 genotypes, we added hypertension as a fourth risk factor for analyses. Interleukin 1 genotypes (Table 2) were generated using TaqMan^®^ chemistry as described previously [10]. The markers chosen were utilized in a previous study that also measured tooth loss as an outcome in contrast to the number of annual dental visits [3]. Various statistical analyses were performed on the data including genetic association analysis by the PLINK software [11], chi-square, Mann-Whitney U, regression, and ANOVA tests to determine significance.

## 5. Conclusions

Individuals with hypertension, diabetes, those who smoke, or have certain interleukin 1 genotypes have higher frequency of tooth loss. Tooth loss is more dramatic in individuals that accumulate more than one of these risk factors.

## References

[1] National Collaborating Centre for Acute Care (2004). NICE Clinical Guidelines, No. 19.

[2] NIDCR’s Surgeon General’s Report. http://www.nidcr.nih.gov/DataStatistics/SurgeonGeneral/.

[3] Giannobile W.V., Braun T.M., Caplis A.K., Doucette-Stamm L., Duff G.W., Kornman K.S. (2013). Patient stratification for preventive care in dentistry. J. Dent. Res..

[4] McGarvey E.L., Leon-Verdin M., Killos L.F., Guterbock T., Cohn W.F. (2011). Health disparities between Appalachian and non-Appalachian counties in Virginia USA. J. Community Health.

[5] Johnston L., Vieira A.R. (2014). Caries experience and overall health status. Oral Health Prev. Dent..

[6] (2002). Status of Oral Health in Pennsylvania.

[7] Huynh-Ba G., Lang N.P., Tonetti M.S., Salvi G.E. (2007). The association of the composite IL-1 genotype with periodontitis progression and/or treatment outcomes: A systematic review. J. Clin. Periodontol..

[8] Steinberger J., Daniels S.R. (2003). Obesity, insulin resistance, diabetes, and cardiovascular risk in children. Circulation.

[9] Vieira A.R. (2014).

[10] Menezes R., Marazita M.L., McHenry T.G., Cooper M.E., Bardi K., Brandon C., Letra A., Martin R.A., Vieira A.R. (2009). AXIN2, orofacial clefts and positive family history for cancer. J. Am. Dent. Assoc..

[11] Purcell S., Neale B., Todd-Brown K., Thomas L., Ferreira M.A., Bender D., Maller J., Sklar P., Bakker P.I., Daly M.J. (2007). PLINK: A tool set for whole-genome association and population-based linkage analyses. Am. J. Hum. Genet..

